# “Low Cost” Pore Expanded SBA-15 Functionalized with Amine Groups Applied to CO_2_ Adsorption

**DOI:** 10.3390/ma8052495

**Published:** 2015-05-11

**Authors:** Enrique Vilarrasa-García, Juan Antonio Cecilia, Elisa Maria Ortigosa Moya, Celio Loureiro Cavalcante, Diana Cristina Silva Azevedo, Enrique Rodríguez-Castellón

**Affiliations:** 1 Department of Chemical Engineering, Universidade Federal do Ceará, Campus do Pici, bl. 709, 60455-760 Fortaleza, Brazil; E-Mails: evilarrasa@uma.es (E.V.-G.); celio@gpsa.ufc.br (C.L.C.); diana@gpsa.ufc.br (D.C.S.A.); 2 Departament of Inorganic Chemistry, Cristallography and Mineralogy, Universidad de Málaga, Campus de Teatinos s/n, 29071 Málaga, Spain; E-Mails: jacecilia@uma.es (J.A.C.); 061776459x@uma.es (E.M.O.M.)

**Keywords:** SBA-15, 1,3,5-triisopropylbenzene, ammonium fluoride, adsorption, CO_2_, 3-aminopropyltriethoxysilane, polyethylenimine ethylenediamine, Dualsite Langmuir model, chemisorptions

## Abstract

The CO_2_ adsorption capacity of different functionalized mesoporous silicas of the SBA-15 type was investigated and the influence of textural properties and the effect of the silicon source on the CO_2_ uptake studied. Several adsorbents based on SBA-15 were synthesized using sodium silicate as silicon source, replacing the commonly used tetraethyl orthosilicate (TEOS). Thus, we synthesized three couples of supports, two at room temperature (RT, RT-F), two hydrothermal (HT, HT-F) and two hydrothermal with addition of swelling agent (1,3,5-triisopropylbenzene) (TiPB, TiPB-F). Within each couple, one of the materials was synthesized with ammonium fluoride (NH_4_F). The supports were functionalized via grafting 3-aminopropyltriethoxysilane (APTES) and via impregnation with polyethylenimine ethylenediamine branched (PEI). The adsorption behavior of the pure materials was described well by the Langmuir model, whereas for the amine-silicas, a Dualsite Langmuir model was applied, which allowed us to qualify and quantify two different adsorption sites. Among the materials synthesized, only the SBA-15 synthesized at room temperatures (RT) improved its properties as an adsorbent with the addition of fluoride when the silicas were functionalized with APTES. The most promising result was the TiPB-F/50PEI silica which at 75 °C and 1 bar CO_2_ captured 2.21 mmol/g.

## 1. Introduction

Since the beginning of the industrialization era, the emissions of greenhouse gases such as carbon dioxide (CO_2_), methane (CH_4_), nitrous oxide (N_2_O), chlorofluorocarbons (CFC), and sulfur hexafluoride (SF_6_) have been continuously increasing. Thus, the average world temperature has increased by 0.74% in the past 100 years and is expected to increase by another 6.4% by the end of the twenty-first century [1]. The emissions of greenhouse gases cause the global warming, which is directly related with droughts, floods, heat waves, and destruction of ecosystems [2]. This fact has led to more restrictive environmental requirements by governments to reduce CO_2_ emissions to the atmosphere.

Among the currently available technologies, post-combustion capture, a technology for capturing CO_2_ from post-combustion emission gases, is the most easily applied technology for existing emission sources. Post-combustion capture uses wet/dry adsorbents, which are used for gas separation, and separates and collects CO_2_ by adsorption/desorption. In general, post-combustion capture technologies include wet absorption, dry adsorption, membrane-based technologies, and cryogenics [3,4].

Wet absorption is good for treating large emission volumes from combustion, however, it demands high energy for absorbent regeneration, suffers from loss of effectiveness over time due to low solvent thermal stability, and usually requires a large footprint. In addition, this technology has some drawbacks such as high equipment corrosion rate and losses due to solvent evaporation [5,6]. Membrane-based processes generally require simple devices, are easy to operate, and have low energy consumption. However, they entail high-cost membrane modules, which are not suitable for treating large volumes of emission gases and may suffer from fast fouling [7,8]. Cryogenics requires low investment cost and it is a mature technology; however, it is not suitable in most cases because of its energy consumption. Current research is focused on dry adsorption systems using dry adsorbents. This technology requires simple devices, which are robust and easy to operate [9]. Nevertheless, the choice of an adsorbent with a suitable working capacity, selectivity, and regenerability is still an open issue.

The process of capturing CO_2_ using a dry adsorbent involves selective separation of CO_2_ based on gas-solid interactions, using molecular sieves such as zeolites [10,11,12], activated carbons [13,14], metal-organic frameworks (MOF) [15,16] and porous silica such as SBA-15 [17,18,19,20,21,22,23,24,25], MCM-41 [21,26,27,28] and mesocellular foams (MCF) [22,24,25,29,30]. The synthesis of both SBA-15 and MCM-41 requires surfactant templates in order to give rise to ordered mesoporous materials. Note that the polymer employed to obtain SBA-15, poly(ethylene oxide)–poly(propylene oxide)–poly(ethylene oxide) (PEO–PPO–PEO), is biodegradable and cheaper than the surfactants used initially in the synthesis of MCM-41 [31]. In addition, a feature of SBA-15 is the interconnection between channels that facilitate diffusion inside the entire porous structure and may pose a significant contribution in gas physisorption [32]. In the last decade, many efforts have focused on the modification of SBA-15 to more effectively tailor it for its application as catalyst or adsorbent.

Thus, the pore size of SBA-15 can be increased by the incorporation of swelling agents such as aromatics compounds [24,25] or alkanes [24], which favors the diffusion of bulky molecules. The presence of fluoride species in the synthesis conditions limits the aggregation and growth of the mesochannels and disrupts the honeycomb arrangement of SBA-15, leading to spherical cells that are interconnected by windows with a narrow pore size distribution [24,25,33]. An important issue related to the potential applications of the ordered mesoporous materials, such as SBA-15 or MCM-41, is their large scale synthesis, which needs to be simple and economically feasible. Alkoxides, such as tetraethyl orthosilicate (TEOS), are a quite expensive silica source, which should be replaced by a less costly and more environmentally friendly silica compound. Stucky *et al.* [34] reported a slightly modified synthesis of SBA-15 using an inexpensive sodium metasilicate as silicon source, obtaining materials with similar behavior as those prepared with TEOS.

The incorporation of amine species (by impregnation or grafting) produces a significant increase in the CO_2_ adsorption capacity. In the case of the impregnation method, large amounts of amine polymers are stabilized inside the porous framework by electrostatic interactions between the amine groups and the silanol species of the porous silica based material [17,24,25,30]. Nonetheless, bulky amine polymers may detrimentally affect CO_2_ adsorption by clogging the porous matrix [35]. The grafting method takes place by reacting an aminosilane the silanol species present on the solid surface, leading to amine modified porous silica. Such grafted silicas have been reported to have high stability and outstanding CO_2_ capacity [20,24,25].

The aim of this work was the synthesis and characterization of SBA-15 using a low cost and environmentally friendly source such as sodium metasilicate. The uniqueness of this study is based on the modification of low-cost SBA-15 with the use of a swelling agent such as 1,3,5-triisopropylbenzene, which increases the pore size and/or the addition of fluoride, which limits the growing of mesochannels of the porous framework, diminishing the diffusion problems. In both cases the textural properties of the low-cost SBA-15 are strongly modified. The porous silica materials were either grafted with 3-(triethoxysilyl)propylamine (APTES) or impregnated with polyethyleneimine (PEI) to be tested for CO_2_ capture.

## 2. Experimental

### 2.1. Materials

The chemicals used to synthesize the various mesoporous silica samples by soft templating were triblock copolymers Pluronic P123 (PEO_20_PPO_70_PEO_20_ average molecular weigth *M*_n_ = 5800 g mol^−1^, Aldrich, St. Louis, MO, USA), sodium silicate solution (Na_2_Si_3_O_7_ with 27% SiO_2_ and 14% NaOH from Aldrich), sodium hydroxide (NaOH, Prolabo, PA, USA) and sulfuric acid (H_2_SO_4_, VWR 95%). In order to enlarge the pore volume and shorten the length of the channels, 1,3,5-triisopropylbenzene (Aldrich 95%) and NH_4_F (Aldrich) were employed, respectively. The functionalization of the thus synthesized silicas was performed with branched polyethylenimine ethylenediamine (PEI) (average molecular weight *M*_n_~600, Aldrich) and 3-aminopropyltriethoxysilane (APTES) (Aldrich 98%), using methanol (Aldrich 99.9%) and toluene (Aldrich 99.5%) as solvents, respectively. The gases employed in this work were He (Air Liquide, Paris, France, 99.99%), N_2_ (Air Liquide 99.9999%) and CO_2_ (Air Liquide 99.998%).

### 2.2. Preparation of Siliceous Porous Structures

**Scheme 1:**
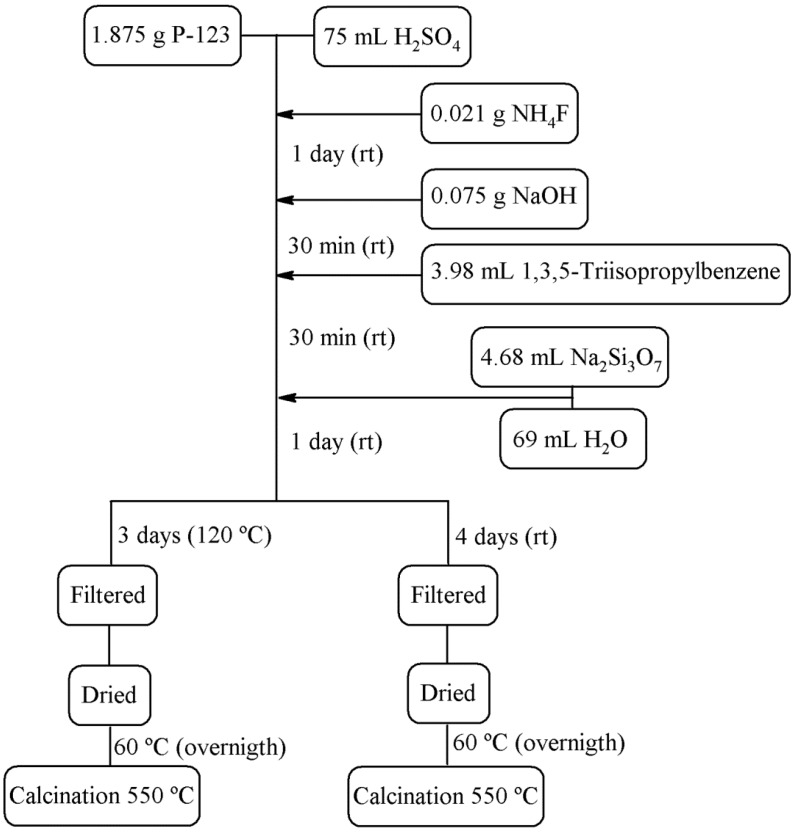
Synthesis of pure silicas.

The synthesis of the low-cost porous silica was obtained following the synthesis reported by Gómez-Cazalilla *et al.* [36] with modifications by the use of a swelling agent such as 1,3,5-triisopropylbenzene or/and NH_4_F to reduce the length of the channels. The synthetic conditions and final molar ratio in the synthesis gel are summarized in Scheme 1 and Table 1.

**Table 1:** Final molar compositon of the synthesis gel.

Sample	Description	P123/SiO_2_/H_2_SO_4_/NaOH/NH_4_F/TIPB/H_2_O
RT	Room temperature SBA15	1/189/93/5.8/0/0/4447
RT-F	Room temperature porous silica modified with fluoride	1/189/93/5.8/1.7/0/4447
HT	Hydrothermal SBA15	1/189/93/5.8/0/0/4447
HT-F	Hydrothermal porous silica modified with fluoride	1/189/93/5.8/1.7/0/4447
TiPB	Hydrothermal SBA-15 modified with pore expander (TiPB)	1/189/93/5.8/0/49/4447
TiPB-F	Hydrothermal porous silica modified with pore expander (TIPB)	1/189/93/5.8/1.7/49/4447

In a typical synthesis, 1.875 g Pluronic was added to 75 mL H_2_SO_4_ 0.4 M solution. After stirring for a few hours, a clear solution was obtained. Then, 0.075 g NaOH and 4.68 mL sodium silicate solution were added at room temperature under magnetic stirring. The resulting gel mixture was stirred for 5 days at room temperature, the final pH was about 1. The solid product was recovered by filtration, washed several times with water, and dried overnight at 60 °C.

All materials were calcined at a heating rate of 1 °C min^−1^ to 550 °C and maintained at this temperature for 6 h. The mesoporous materials were labeled as indicated in Table 1.

### 2.3. Characterization Techniques

X-ray diffraction (XRD) was used to identify the crystalline phases of the synthesized solids. These experiments were performed on an X’Pert Pro MPD automated diffractometer (PANalytical, Almelo, The Netherlands) equipped with a Ge (111) primary monochromator (strictly monochromatic Cu-Kα radiation) and an X’Celerator detector. Measurements were obtained for 2 h in the range of 1°–10°.

The textural parameters (S_BET_, V_P_ and D_P_) were evaluated from nitrogen adsorption–desorption isotherms at −196 °C as determined by an automatic ASAP 2020 system from Micromeritics. Prior to the measurements, samples were degassed at 200 °C and 10^−4^ mbar overnight. Surface areas were determined by using the Brunauer–Emmett–Teller (BET) equation assuming a cross section of 16.2 Å^2^ for the nitrogen molecule. Micropore surface areas were obtained by de Boer’s t-plot method [37] for relative pressures between 0.35 and 0.70. The pore size distribution was calculated by applying the Barrett–Joyner–Halenda (BJH) method applied to the desorption branch of the N_2_ isotherm. The total pore volume was calculated from adsorbed N_2_ at *P*/*P*_0_ = 0.996

FTIR spectra were collected on a Varian 3100 FTIR spectrophotometer (Varian Inc., Walnot Creek, CA, USA). The interferograms consisted of 200 scans, and the spectra were collected using a KBr spectrum as background. About 30 mg of a mixture of each sample and KBr at a weight ratio of 1:9 were placed in the sample holder and then spectra were collected.

Elemental chemical analysis was performed with a LECO CHNS 932 analyzer (LECO Corporation, St. Joseph, MI, USA) to determine the nitrogen content present through the combustion of the samples at 1100 °C in pure oxygen to form NO.

Thermal analysis was performed on a TG-DTG thermobalance (Mettler Toledo, Columbus, OH, USA) with a continuous heating rate of 5 °C min^−1^ in a helium flow.

### 2.4. CO_2_ Adsorption Tests

CO_2_ adsorption isotherms were measured with a Micromeritics ASAP 2020 Analyzer (Micromeritics Corp., Norcross, GA, USA) (*i.e.*, volumetrically) at 25 °C. Prior to the measurements, samples were degassed at 115 °C and 10^−4^ mbar overnight.

## 3. Results and Discussion

### 3.1. Characterization

**Figure 1:**
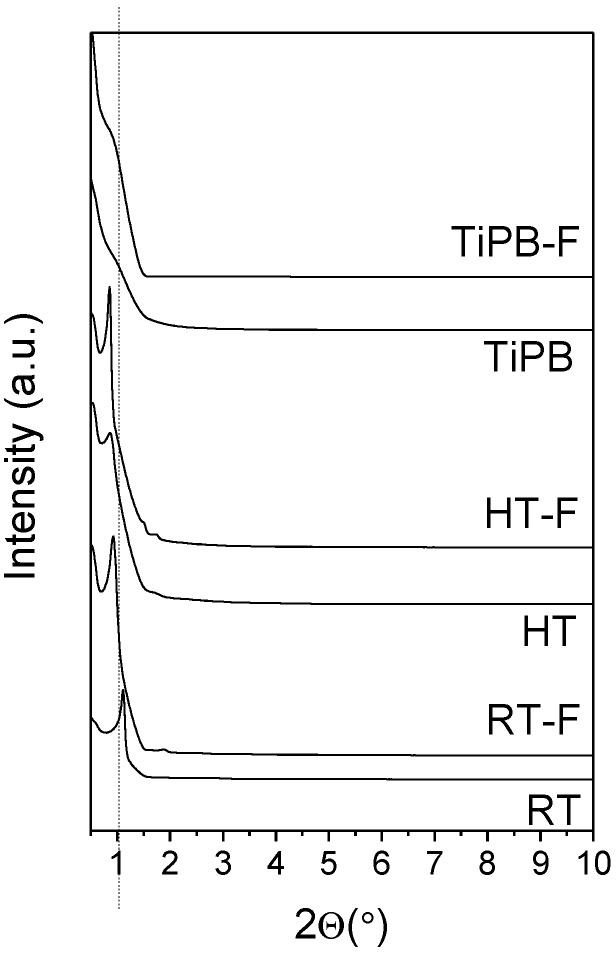
Low angle X-ray diffraction (XRD) patterns of pure silicas.

The low-angle XRD diffractograms of the synthesized samples are depicted in Figure 1. Samples RT, RT-F, HT and HT-F (samples are labeled as indicated in Table 1) clearly show the (100) reflection, which is associated to the P6mm hexagonal arrangement [38]. Although the addition to fluoride limits the length of the mesochannels, there is a shift of the (100) reflection towards lower values, which suggests an increase of the pore size in the hexagonal framework in all cases. The hydrothermal treatment leads to a shift to even lower 2θ(°) values, revealing the formation of better ordered hexagonal materials with a larger pore size.

The addition of a swelling agent, such as 1,3,5-triisopropylbenzene, leads to a shift of the (100) reflection to lower angles, which has been attributed to the incorporation of swelling agents such as alkanes or derived benzene compounds that penetrate into the hydrophobic core of the surfactant micelle of P-123, disrupting the honeycomb packing typical of the hydrothermal SBA-15, leading to node separation into spherical micelles [39,40].

**Figure 2:**
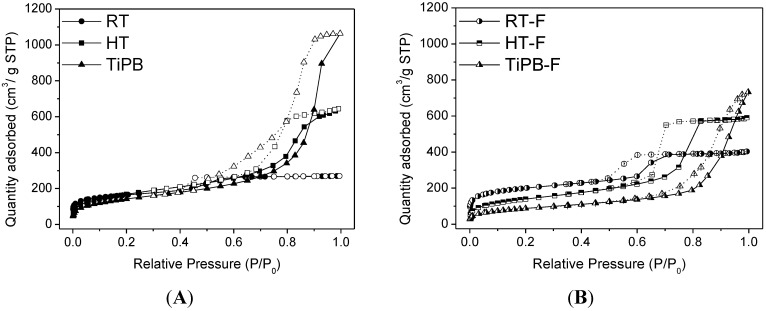
Nitrogen adsorption/desorption isotherms at −196 °C of pure silicas: (**A**) without fluoride (**B**) with fluoride.

Figure 2 shows the nitrogen adsorption/desorption isotherms of the porous silicas. Samples without fluoride (RT, HT and TiPB) exhibit the classic type IV isotherms with parallel H1 type hysteresis loops. The hysteresis loop, which accounts for capillary condensation, is shifted toward higher *P*/*P*_0_ when a swelling agent is added (TiPB). This fact is related to the larger pore sizes by the addition of 1,3,5-triisopropylbenzene, which is known to widen the hydrophobic core of the surfactant that shapes the pores of the silica.

The addition of ammonium fluoride also causes a shift of hysteresis loop to higher relative pressure together with a decrease in surface area, except for RT and RT-F samples, in which case the surface area increases with the addition of the fluoride salt (Table 2). This fact suggests that the temperature synthesis could have an influence on the behavior of samples. (RT and RT-F were synthesized at 25 °C).

Table 2 summarizes the textural properties of pure silicas. Note that the less ordered materials present lower surface areas but exhibit higher pore volumes. The addition of swelling agent produced the desired effect, leading to pore expanded materials (7.9 and 13.2 nm for TiPB and TiPB-F, respectively). Micropore volume and micropore surface area were higher for more ordered materials. In these materials, the micropores are formed due to the hydrophilic character of the ethylene oxide chains, which remain trapped within the siliceous walls during the condensation step and leave interconnecting micropores upon surfactant removal by calcinations or solvent extraction [32,41,42,43,44].

**Table 2:** Textural properties of pure silicas.

Sample	*S*_BET_ (m^2^/g)	*S*_mp t.plot_ (m^2^/g)	*V*_P_ (cm^3^/g)	*V*_mp t-plot_ (cm^3^/g)	*D*_P_ (nm)
RT	571	256	0.42	0.12	3.6
RT-F	699	385	0.62	0.17	4.6
HT	613	148	0.99	0.06	7.5
HT-F	508	74	0.91	0.03	6.3
TiPB	519	74	1.64	0.02	7.9
TiPB-F	321	75	1.13	0.02	13.2

Notes: *S*_BET_: equivalent surface area, as determined by Brunauer-Emmett-Teller equation; *S*_mp t.plot_/*V*_mp t-plot_: micropore surface area/micropore volume area, as determined by t-plot method; *V*_P_: total pore volume, as calculated from adsorbed N_2_ at *P*/*P*_0_ = 0.996 (−196 °C); *D*_P_: pore diameter, as calculated by Barret-Joyner-Halenda (BJH) method to the desorption branch of the N_2_ isotherm at −196 °C.

**Figure 3:**
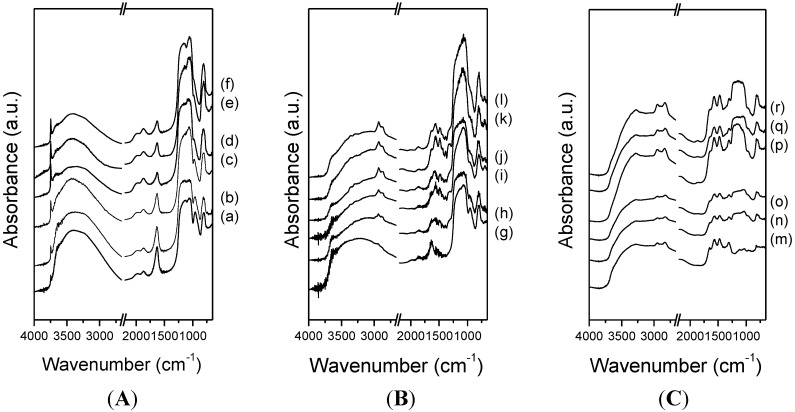
FTIR spectra of (**A**) pure silicas; (**B**) amine grafted silicas and (**C**) amine impregnated silicas (a) RT; (b) RT-F; (c) HT, (d) HT-F; (e) TiPB, (f) TiPB-F; (g) RT/20A, (h) RT-F/20A; (i) HT/20A; (j) HT-F/20A; (k) TiPB/20A; (l) TiPB-F/20A; (m) RT/50P; (n) RT-F/50P; (o) HT/50P; (p) HT-F/50P; (q) TiPB/50P and (r) TiPB-F/50P.

The FTIR spectra for the different synthesized pure silicas and their respective amine functionalized counterparts are shown in Figure 3. The FTIR spectra of pure silicas (Figure 3A) show a narrow and intense band around 3700 cm^−1^ and a broad low frequency band centered at 3400 cm^−1^. A first band is related to the symmetrical stretching vibration mode of isolated O-H in terminal silanol groups [45] and the broad band centered at 3400 cm^−1^ is due to those silanol groups with cross hydrogen bonding interactions with adsorbed water molecules [20,27,46,47,48]. The above mentioned band at around 3700 cm^−1^ has a major importance to ensure high grafting efficiency of the amine groups.

The spectra of amine silicas (Figure 3B,C) reveal the presence of two bands at 2950 and 2840 cm^−1^ assigned to C-H asymmetric and symmetric stretching modes of the APTES and PEI chains, respectively [49]. Furthermore, the bands located at 1565 and 1485 cm^−1^ are attributed to asymmetric and symmetric bending of primary amines (-NH_2_) [25,50]. The presence of two bands around 3300 cm^−1^ is noticeable and may be assigned to N-H stretching vibrations [25], which provides evidence for the successful APTES grafting of the porous silicas [51,52]. Finally, the intensity of the band mentioned above, at around 3700 cm^−1^, decreases or is not observed in the FTIR spectra of amine functionalized silicas. This suggests that surface silanol groups have reacted with ethoxy groups from the aminosilane, thus providing evidence of the incorporation of organic molecules in the porous silica materials.

It is noteworthy that the band located at 1054 cm^−1^ related to Si-O-Si asymmetric stretching with a shoulder at 1160 cm^−1^ appears with a strong intensity in the amine grafted silicas (Figure 3B) whereas it is weaker in the more ordered impregnated silicas, perhaps due to the deposition of PEI layers that prevents a clean visualization of these bands.

### 3.2. CO_2_ Adsorption

**Figure 4:**
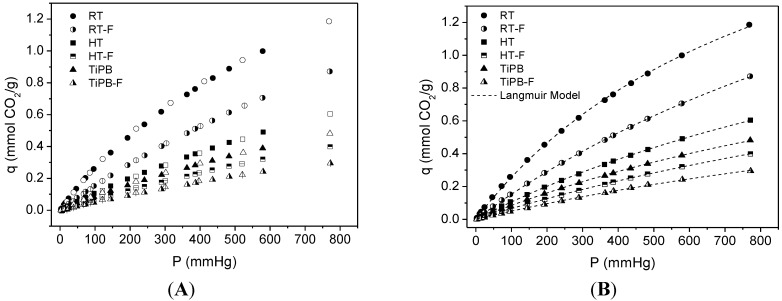
(**A**) CO_2_ adsorption/desorption isotherms at 25 °C on pure silicas. Filled symbols stand for adsorption data and empty symbols stand for desorption data; dotted lines (**B**) are fits from the Langmuir model equation using adsorption data.

Figure 4 shows CO_2_ adsorption /desorption isotherms on pure silicas obtained with sodium silicate solution. For non-functionalized supports, adsorption isotherms are virtually linear, thereby all isotherms show a gradual increase in the amount of the adsorbed CO_2_ with an increase of pressure. Except for the samples synthesized at room temperature (RT and RT-F), all silicas adsorbed CO_2_ at less than 0.6 mmol/g.

Experimental data were fit to the Langmuir adsorption model. More ordered pure silicas exhibit higher CO_2_ capacities and also higher affinity to CO_2_ capture (*q* and *K* values, Table 3). These results can be associated with the larger microporous network (less ordered materials present lower k values). The isotherms showed a nearly linear behavior, suggesting weak adsorbent–adsorbate affinity.

**Figure 5:**
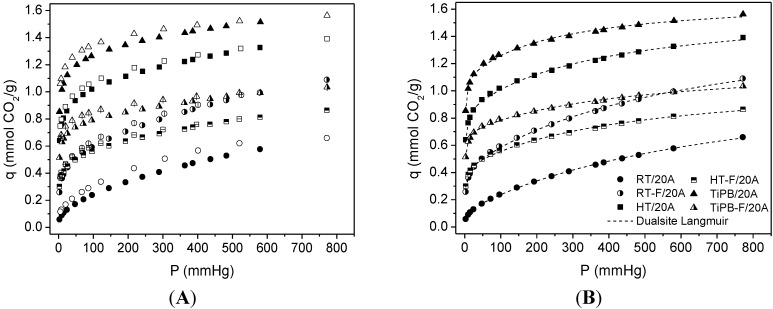
(**A**) CO_2_ isotherms at 25 °C on 3-aminopropyltriethoxysilane (APTES)-grafted silicas Adsorption data are the filled symbols and desorption data are the empty symbols; (**B**) dotted lines are fits from de Dualsite Langmuir model using adsorption data.

In Figure 5, CO_2_ adsorption/desorption isotherms at 25 °C on amine-grafted silicas are plotted. All isotherms present hysteresis, due to strong interactions between CO_2_ and amine groups, leading to incomplete desorption by only decreasing pressure. Note that special care was taken in these experiments to allow for enough time for equilibrium to be reached, so that the hypothesis of diffusional resistances in the adsorption branch as a cause of this hysteresis could be ruled out.

**Table 3:** Parameters from fit to Langmuir model for pure silicas.

Sample	Langmuir Model		*q* (25 °C, 1 bar)
	*q* (mmol CO_2_/g)	*K* (mmHg^−1^)	
RT	2.53 ± 0.05	0.0011 ± 3.4 × 10^−4^	1.19
RT-F	2.88 ± 0.04	5.61 × 10^−4^ ± 1.03 × 10^−5^	0.87
HT	1.99 ± 0.03	5.59 × 10^−4^ ± 1.23 × 10^−5^	0.61
HT-F	1.69 ± 0.04	4.08 × 10^−4^ ± 1.06 × 10^−5^	0.39
TiPB	1.58 ± 0.03	5.66 × 10^−4^ ± 1.59 × 10^−5^	0.48
TiPB-F	1.16 ± 0.06	4.47 × 10^−4^ ± 2.73 × 10^−5^	0.29

**Figure 6:**
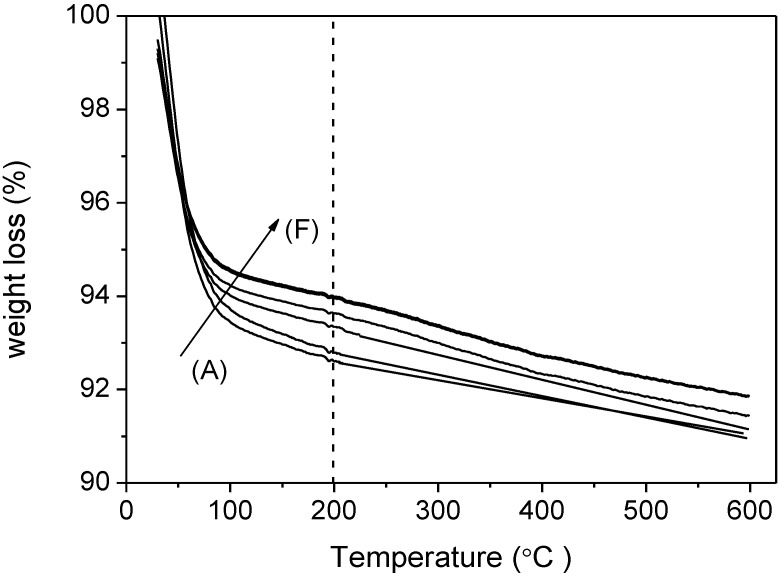
Thermogravimetry (TGA) thermograms of (A) RT; (B) RT-F; (C) HT; (D) HT-F; (E) TiPB and (F) TiPB-F.

All CO_2_ adsorption isotherms showed an initial steep slope at pressures below 100 mmHg, followed by a smooth increase in the amount of the adsorbed CO_2_ from this pressure up. This behavior was also observed in impregnated silicas (see Figure 6). The nature of this steep rise can be related to the chemical reaction between CO_2_ and amine groups, and the subsequent gradual increase in the amount of the adsorbed CO_2_ attributed to CO_2_ adsorbed by physisorption. In the case of high amine density, e.g., the impregnation with PEI, the CO_2_ adsorbed interacts by hydrogen bonding with neighboring amine sites and promotes the formation of strongly adsorbed CO_2_. This fact is confirmed by the high adsorption at lower absolute pressure. However, for low amine density, e.g., grafting with APTES, the CO_2_ species lack neighboring amine sites and bind weakly adsorbed CO_2_, so these sites can be easily removed; the chemisorptions sites of the materials functionalized with APTES are less strong than those functionalized with PEI [22,23]. From this reasoning and based on previous works [24,25], we used the Dualsite Langmuir model (DsL) (Equation (1)) to describe isotherms of functionalized materials with the aim to distinguish between the contributions due to chemical reaction and physical interactions.

(1) $$q=\frac{q_{1}K_{1}P}{1+K_{1}P}+\frac{q_{2}K_{2}P}{1+K_{2}P}$$

Site 1 was taken as that with higher *k* parameter and is hence assigned as a chemisorption site whereas site 2 accounts for physical adsorption. The fitted parameters are presented in Table 4. It is noteworthy that the more ordered silicas showed higher CO_2_ capacities than the pore expanded materials, in terms of physisorption (see *q*_2_ values in Table 4), following the same trend observed in pure silicas. Despite the fact that the presence of a more extended microporous structure enhances physical adsorption of CO_2_, the pore expanded materials show a higher amount of chemisorbed CO_2_, because these materials have higher grafting yields, in agreement with elemental analysis results. APTES surface coverage [25,53], as calculated by Equation (2), is also included in Table 4.

(2) $$\%APTES\;surface\;coverage=\frac{molar\;N\;content\;\times \;2\;\times \;N_{A}}{3.7\;\times \;S_{BET}}\;\times \;100$$

where *N* is the molar concentration of nitrogen (mol g^−1^), as determined by elemental analysis, 2 accounts for the assumption that two surface silanol groups react with two ethoxy groups of each APTES molecule, in accordance with Chang *et al.* [54] who proposed bidentate coordination. *S*_BET_ is the BET surface area of amine-grafted silica (m^2^g^−1^) and 3.7 is the density of silanol groups per nm^2^, as determined by Shenderovich *et al.* [55] through Si-NMR analysis for this class of materials. Although not necessarily these materials present 3.7 as silanol density, for comparison purposes we can use that density as all materials synthesized in this work exhibit a similar silanol density. The silanol densities were estimated by thermogravimetry (TGA). In Figure 6, we can observe that the total weight losses of our pure adsorbents are in the range 8.1%–9.0%, consisting of two mass loss steps, below 200 °C attributed to the removal of the physically and the chemically adsorbed water and the weight loss above 200 °C is related to dehydroxylation by condensation of silanol groups. Due to the weight loss associated with the dehydroxylation being similar (around 2%) for all adsorbents, and from this value being related to the density of silanols [56], these results lead us to consider that the addition of swelling agents has only a slight effect on the silanol densities. However, solvent extraction as a template removal method could be an efficient method to increase this value [56].

**Table 4:** Parameters from fit to Dualsite Langmuir model for amine grafting samples (20% APTES).

Sample	*S*_BET_	%N	%APTES	Dualsite Langmuir model				q*
				*q*_1_	*K*_1_	*q*_2_	*K*_2_	
RT	22	1.37	144	0.12 ± 0.01	0.247 ± 0.045	1.07 ± 0.03	1.31 × 10^−3^ ± 9.95 × 10^−4^	0.66
RT-F	102	2.85	65	0.43 ± 0.01	0.538 ± 0.071	1.17 ± 0.06	1.65 × 10^−3^ ± 1.82 × 10^−4^	1.09
HT	245	3.36	32	0.85 ± 0.01	0.984 ± 0.111	0.77 ± 0.04	2.77 × 10^−3^ ± 3.89 × 10^−4^	1.39
HT-F	260	2.41	21	0.44 ± 0.01	0.727 ± 0.079	0.66 ± 0.03	2.25 × 10^−3^ ± 2.61 × 10^−4^	0.86
TiPB	135	3.89	67	1.09 ± 0.02	1.449 ± 0.148	0.59 ± 0.02	4.16 × 10^−3^ ± 6.22 × 10^−4^	1.56
TiPB-F	198	2.70	32	0.69 ± 0.01	1.107 ± 0.099	0.52 ± 0.03	2.30 × 10^−3^ ± 3.55 × 10^−4^	1.03

Notes: *S*_BET_: equivalent surface area, as determined by Brunauer-Emmett-Teller equation (m^2^/g); %N: N content (wt%) as obtained by chemical elemental analysis (CNH); *q*_x_: mmol CO_2_/g; q*: mmol CO_2_/g at 25 °C and 1 bar; *K*_x_: mmHg^−1^.

The %APTES values are summarized in Table 4. Note that the silanol density was fixed at 3.7 per nm^2^, which led to grafting yields over 100% (RT sample). It is likely that this sample contains a higher silanol density, above 3.7. However, only 1.37% N content was bonded on the RT sample surface. This could be due to the APTES molecules experiencing higher diffusional resistances through the narrow and long channels of the RT sample. This phenomenon is not expected to occur in less ordered materials, which explains the higher %N contents leading to higher CO_2_ adsorption capacities.

In accord with previous works [27,57], primary and secondary amines (an APTES molecule has one primary amine) react with CO_2_ through a zwitterion mechanism where water or amine group is needed (Equation (3))

2(RNH_2_) + CO_2_→RNHCO_2_^−^RNH_3_^+^ (3)

Consequently, under our work conditions (dry conditions), two amine groups are required to capture one CO_2_ molecule, so that the maximum CO_2_/N adsorption efficiency is 0.5. Lower efficiencies may be due to the lack of two amine groups sufficiently close to each other so as to allow reaction and formation of carbamate [58].

In addition, recently others authors have reported that the increase in CO_2_ capture capacity of the N-containing materials could be also attributed to hydrogen bonding interactions between hydrogen atoms (from C-H and N-H) and CO_2_ molecules [59,60], providing a new mechanism of the interaction between CO_2_ and N-doped materials.

The best CO_2_ capacity at 25 °C and 1 bar in this work was 1.56 mmol CO_2_/g or 7 wt%, found in the case of the TPiB silica sample. This value is in the range of those reported by other authors [25,57,61].

**Figure 7:**
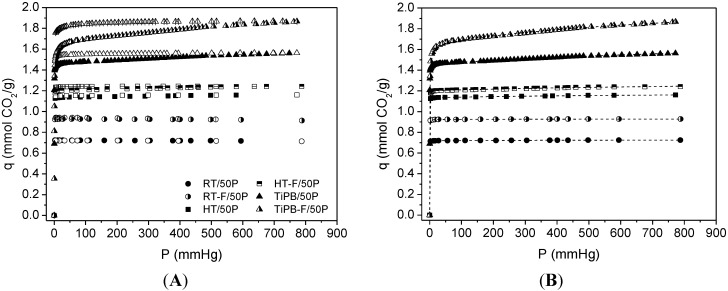
(**A**) CO_2_ isotherms at 25 °C on polyethylenimine ethylenediamine branched (PEI)-impregnated silicas Adsorption data are the filled symbols and desorption data are the empty symbols; (**B**) dotted lines are fits from de Dualsite Langmuir model using adsorption data.

**Figure 8:**
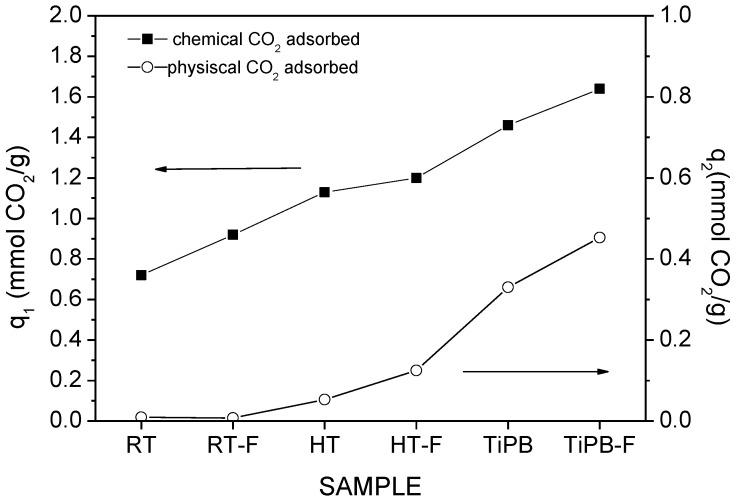
Evolution *q*_1_ and *q*_2_ PEI-silicas.

Figure 7 shows adsorption/desorption CO_2_ isotherms at 25 °C for impregnated silicas (50 wt% PEI). The presence of amine groups on the silicas surface, as observed in amine grafted silicas isotherms, causes a steep rise at low pressures. An outstanding CO_2_ capacity is observed for the TiPB-F sample, reaching 1.87 mmol CO_2_/g at 25 °C and 1 bar. All adsorption isotherms were fitted to the DsL model and the fit parameters are summarized in Table 5. For PEI impregnated silicas, we observed a gradual increase in both chemical and physical CO_2_ adsorption (see *q*_1_ and *q*_2_ values and see also Figure 8). It seems that the evolution to the less ordered materials with expanded pores delays the pore blockage at higher PEI loadings. Further, the MCF type of arrangement improves the accessibility to amine groups, as we observed in previous works [24].

Upon loading of polyethylenimine up to a certain level, the porous structure of the original supports remained unchanged, although the surface area and pore volume decreased drastically. Accordingly, CO_2_ adsorption capacities of the resulting adsorbents increased with the loading of PEI up to a maximum loading value, beyond which over-loading of the amines had a detrimental effect on CO_2_ capture capacities.

**Table 5:** Parameters from fit to Dualsite Langmuir model for amine impregnated samples (50% polyethylenimine ethylenediamine branched (PEI)).

Sample	*S*_BET_	%N	Dualsite Langmuir model				q*
			*q*_1_	*K*_1_	*q*_2_	*K*_2_	
RT	40	15.81	0.72 ± 0.01	56.051 ± 5.095	9.21 × 10^−3^ ± 1.13 × 10^−3^	2.31 × 10^−3^ ± 7.40 × 10^−4^	0.72
RT-F	81	15.07	0.92 ± 0.01	35.113 ± 2.874	7.29 × 10^−3^ ± 1.91 × 10^−3^	2.33 × 10^−3^ ± 1.58 × 10^−3^	0.93
HT	103	15.23	1.13 ± 0.01	42.840 ± 5.312	0.05 ± 0.01	1.32 × 10^−3^ ± 2.64 × 10^−4^	1.16
HT-F	101	14.18	1.20 ± 0.01	19.234 ± 1.299	0.12 ± 0.02	6.53 × 10^−4^ ± 1.51 × 10^−4^	1.24
TiPB	146	13.62	1.46 ± 0.01	11.783 ± 0.028	0.33 ± 0.03	5.95 × 10^−4^ ± 7.19 × 10^−5^	1.56
TiPB-F	171	14.63	1.64 ± 0.01	2.795 ± 0.069	0.45 ± 0.03	1.12 × 10^−4^ ± 1.35 × 10^−5^	1.87

Notes: *S*_BET_: equivalent surface area, as determined by Brunauer-Emmett-Teller equation (m^2^/g); %N: N content (wt%) as obtained by chemical elemental analysis (CNH); *q*_x_: mmol CO_2_/g; q*: mmol CO_2_/g at 25 °C and 1 bar; *K*_x_: mmHg^−1^.

It is believed that under extreme high amine loadings, all the pores are filled and the external surface of SBA-15 will also be coated by the sticky amines, leading to limited accessibility of CO_2_ towards the bulk amine molecules [62,63]. According to this reasoning, the maximum amount of amines that a mesoporous silica support can accommodate depends upon its pore volume. This hypothesis has been verified by experimental observation that when the volume corresponding to the amount of impregnated amine exceeds the total pore volume of the support, the resulting adsorbent appears to be sticky and gel-like. The high viscosity of the organic amines will also cause difficulties in CO_2_ diffusion from the surface to the bulk amines, therefore a two-stage adsorption kinetics was observed (a fast surface reaction stage followed by a slower diffusion-controlling stage) [64,65], and where it appears that rather than lower temperatures, the highest capacities were typically obtained at 75 °C [52], so as to get rid of the diffusion barrier.

**Figure 9:**
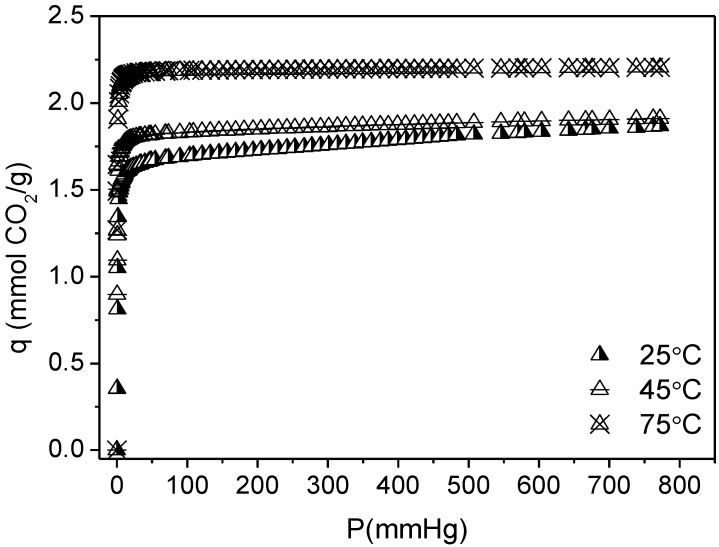
CO_2_ adsorption isotherms for TiPB-F/50 PEI at 25, 45 and 75 °C.

**Figure 10:**
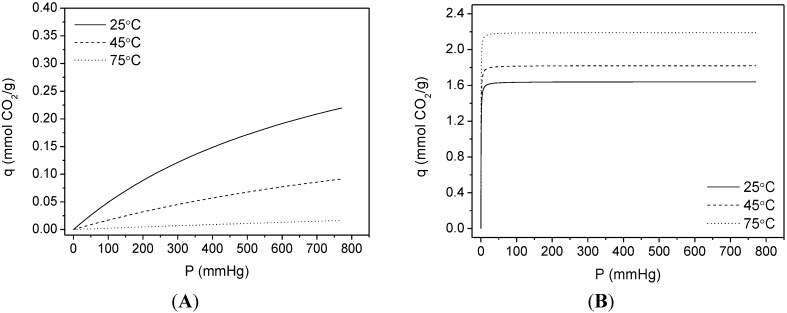
Deconvolution of CO_2_ adsorption isotherms for TiPB-F/50PEI in their respective physisorption (**A**) and chemisorption (**B**) contributions.

The sample that achieved the higher CO_2_ capacity (TiPB-F/50 PEI) was also tested at 45 and 75 °C and CO_2_ adsorption isotherms are shown in Figure 9. CO_2_ capacity increases at higher temperatures, as expected. TiPB-F/50 PEI reached 1.87, 1.91 and 2.21 mmol CO_2_/g at 25, 45 and 75 °C, respectively. This increase in capacity at higher temperatures is due to chemisorption gain, as shown in Figure 10. The increase of the *K*_1_ values (2.74, 3.25 and 4.61 mmHg^−1^ for 25, 45 and 75 °C respectively) from DsL fit with temperature rise signifies that the process needs thermal energy, meaning that the process is endothermic. This is consistent with the assumption that chemisorptions occurs in site 1 [66]. SBA-15 synthesized from sodium silicate solution is a feasible option to obtain mesoporous silica with interesting capacities of CO_2_ adsorption and probably with high CO_2_/N_2_ selectivity, especially SBA-15 impregnated and working at 75 °C, where the chemisorptions process prevails.

## 4. Conclusions

Several mesoporous silicas were synthesized using sodium silicate, a cheaper silicon and environmentally benign source than traditional silicon alkoxides. The addition of swelling agents (1,3,5-triisopropylbenzene) and a solubility enhancer (ammonium fluoride) succeeded in producing materials with different textural features, but the addition of ammonium fluoride was not beneficial because it led to materials with lower pore volume as compared to those from the same synthesis performed without ammonium fluoride.

The materials were functionalized by grafting with APTES (20% v/v) and by impregnation with PEI (50 wt%) and the CO_2_ adsorption /desorption isotherms were measured. Materials with APTES had a direct relationship between the capacity and surface area of the non-functionalized material.

Among the materials synthesized, only SBA-15 synthesized at room temperatures (RT) improved its properties as an adsorbent with the addition of fluoride. The other solids showed poorer performance with the fluoride presence. The most promising result was the mesoporous silica synthesized with TIPB without fluoride (TIPB) at 25 °C and 1 bar CO_2_ which captured 1.56 mmol/g (69 mg CO_2_/g).

In the case of materials impregnated with PEI, the addition of fluoride was beneficial when synthesized with sodium silicate, the twisting up of the structure causes better performance of the material in terms of CO_2_ adsorption, reaching 2.21 mmol CO_2_/g at 75 °C and 1 bar. Therefore, compared with the results obtained in others works, sodium silicate can replace TEOS with the consequent reduction in the cost without the material losing potential properties as a CO_2_ adsorbent.
